# Enzalutamide-Induced Upregulation of PCAT6 Promotes Prostate Cancer Neuroendocrine Differentiation by Regulating miR-326/HNRNPA2B1 Axis

**DOI:** 10.3389/fonc.2021.650054

**Published:** 2021-06-30

**Authors:** Bo Liu, Hui-Yang Jiang, Tao Yuan, Jie Luo, Wei-Dong Zhou, Qi-Quan Jiang, Denglong Wu

**Affiliations:** Department of Urology, Tongji Hospital, Tongji University of Medicine, Shanghai, China

**Keywords:** prostate cancer, neuroendocrine differentiation, PCAT6, miR-326, HNRNPA2B1

## Abstract

Our previous studies have demonstrated that Enzalutamide-induced upregulation of long non-coding RNA p21 (lncRNA-p21) facilitates prostate cancer (PCa) neuroendocrine differentiation (NED). Given the important role of lncRNAs in PCa pathogenesis, and given that lots of lncRNAs are dys-regulated in neuroendocrine PCa (NEPC) patients, we next explored the biological function and underlying mechanism of lncRNA-PCAT6 (PCAT6) in mediating Enzalutamide-induced NED. The level of PCAT6 in Enzalutamide-treated PCa cells and NEPC samples were assessed using quantitative RT-PCR (qPCR). The effect of PCAT6 on PCa cell proliferation, invasion, and NED was evaluated through CCK-8, transwell, qPCR, western blot analysis, Xenograft mouse model, and *in vivo* lung metastasis model. We found that PCAT6 was highly expressed in NE-like cells (PC3, DU145, and NCI-H660) compared with androgen-sensitive LNCaP cells. PCAT6 was also highly expressed in NEPC tissues. Enzalutamide treatment resulted in a significant increase of PCAT6 level in a dose- and time-dependent fashion. Functionally, PCAT6 overexpression promoted NED of C4-2 cells, as evidenced by an increased expression of NE markers (NSE, ChgA, and SYP), whereas PCAT6 knockdown in NCI-H661 cells repressed NED. Furthermore, PCAT6 overexpression promoted PCa cell proliferation and invasion *in vitro* and *in vivo*. Mechanistically, PCAT6 functioned as competing endogenous (ce) RNA *via* absorbing miR-326, thus resulting in a de-suppression of *Hnrnpa2b1* target gene. The current results demonstrate that PCAT6 acted as a tumor activator in PCa progression by sponging miR-326 and increasing Hnrnpa2b1 expression and that the PCAT6/miR-326/Hnrnpa2b1 signaling might be a new therapeutic target for PCa.

## Introduction

Prostate cancer (PCa) is the second most common male cancer globally and has a high mortality (1). For men diagnosed with localized PCa, surgical operation and radiotherapy are the most effective treatment options. Significant technological advances have been made in surgical operation and radiotherapy, such as robotic surgery as well as 3D-confromal radiotherapy (2). Androgen deprivation therapy (ADT) is the main therapeutic for advanced PCa (3). Currently, the most widely used anti-androgen drugs are bicalutamide, Abiraterone, and Enzalutamide (4). To some extent, Enzalutamide treatment prolongs the survival time of PCa patients, but Enzalutamide would also lead to a series of side effects, including drug resistance, cancer metastasis, and PCa NED (5, 6). However, the mechanism of underlying Enzalutamide-induced PCa NED remains unclear.

Long noncoding RNAs (lncRNAs) are a class of non-coding RNA with a transcript longer than 200 nt and not encoded proteins themselves, but can regulate gene expression (7). Recent studies have shown that lncRNAs play a critical role in different types of physiopathologic processes, such as embryonic development, organ formation, tumorigenesis, and tumor progression (8, 9). Abnormal lncRNAs exert crucial roles in regulating cancer cell proliferation and invasion (10). Prostate cancer-associated transcript 6 (PCAT6) has been demonstrated to play a cancer-promoting role in various cancers, including gastric cancer (11), lung cancer (12), as well as cervical cancer (13). PCAT6 is abnormally overexpressed in PCa, and promotes tumor cell proliferation as well as invasion in an androgen-independent way (14, 15). However, the potential function of PCAT6 on PCa NED remains unclear.

Heterogeneous nuclear ribonucleoproteins (hnRNPs) are a class of RNA-binding proteins that form hnRNP-RNA complexes by binding to precursor mRNA (pre-mRNA) and subsequently participating in the splicing, translation, transport, and degradation of mRNA (16). HnRNPA2B1 is a crucial member of the hnRNP family, and is correlated with tumorigenesis and the progression of breast cancer, pancreatic cancer, and glioblastoma (17, 18). Yin et al., demonstrated that upregulated hnRNPA2B1 accelerates gliomas cell growth through activating signal transducer and activator of transcription 3 (STAT3) and AKT signaling pathways (18).

HnRNPA2B1 also possesses pro-oncogenic functions in glioblastoma, and low expression of hnRNPA2B1 predicts a better prognosis in patients with glioblastoma (19). However, the role of hnRNPA2B1 in PCa NED remains unclear.

Herein, the biological roles and underlying mechanisms of PCAT6 in Enzalutamide-induced PCa NED were explored. We demonstrated that PCAT6 was highly expressed in NE-like cell lines and NEPC tissues. Enzalutamide treatment enhanced PCAT6 level in a dose- and time-dependent fashion. Overexpression of PCAT6 facilitated NED of C4-2 cells, whereas PCAT6 knockdown in NCI-H661 cells repressed NED. PCAT6 acted as ceRNA through absorbing miR-326, thus resulting in a de-suppression of hnRNPA2B1.

## Materials and Methods

### Patient Sample

PCa tissues and matched normal tissues were collected from Tongji Hospital, Tongji University School of Medicine. The protocols used in this research were approved by the Hospital’s Protection of Human Subjects Committee and written informed consent was provided by all patients or their legal guardians.

### Cell Culture

NE-like cells (PC3, DU145, and NCI-H660), LNCaP, C4-2 cells were purchased from ScienCell (Santiago, California, USA). All cells were maintained in RPMI-1640 media containing 10% FBS at 37°C in a 5% CO_2_ incubator.

### Overexpression and RNA Interference (RNAi)

MiR-326 mimic (miR-326), mimic control (miRcont), siRNA for PCAT6 (si- PCAT6), hnRNPA2B1 (si-hnRNPA2B1), and scrambled siRNA (si-NC) were obtained from Shanghai Genechem Co., LTD (Shanghai, China). To overexpress PCAT6, human PCAT6 (NR_046326.1) cDNA was cloned into the pcDNA3.1 vector (Invitrogen). si-PCAT6, UGGCCUAGGAACCCGA ACCUGACCC; si-hnRNPA2B1, 5’-GGAACAGUUCCGUAAGCUC-3’; miR-326 mimic, 5’-GCAGGGCACGACUGAUCUUGG-3’.

### RNA Extraction and Quantitative Real-Time PCR (qPCR)

Total RNA was extracted from PCa tissues cells with Trizol reagent (Takara, Japan). Special miRNA primers were applied for the reverse transcription (RT) of miRNAs. Oligo (dT) and random primers were applied for the RT of mRNA and lncRNA. qPCR was carried out by SYBR Green qPCR Master Mix (APExBIO, Houston, USA) on a Bio-Rad CFX96 system (BioRad, Hercules, CA, USA) following the manufacturer’s protocol. Related genes expression levels were normalized to GAPDH expression. The fold change was calculated through the 2^-ΔΔCt^ method. miR-326, Forward 5’-CCTCTGGGCCCTTCCTCCAG-3’, Reverse 5’-CTGGAGGAAGGGCCCAGAGG-3’; PCAT6, Forward 5’- CCCCTCCTTACTCTTGGACAAC-3’, Reverse 5’-GACCGAATGAGGATGG AGACAC-3’; NES, Forward 5’-GCAGAGGTCTACCACACACTCAA-3’, Reverse 5’-GGCTTCCTTCACCAGTTCCA-3’; SYP, Forward 5’-CTTTGCCATCTTCGCCTTT-3’, Reverse 5’-AGGTCACTCTTGGTCTTGTTGG-3’; CHgA, Forward 5’-TACAAGGAGATCCGGAAAGG-3’, Reverse 5’-CCATCTCCTCCTCCTCCTCT-3’; GAPDH, Forward 5’-TGAAGGGTGGAGCCAAAAG-3’, Reverse 5’-AGTCTTCTGGGTGGCAGTGAT-3’.

### Fluorescent In Situ Hybridization (FISH)

The expression and localization of PCAT6 were detected with FISH by RiboTM lncRNA FISH Probe Mix (Green) (RIBOBIO, Guangzhou, China). LNCaP and NCI-H660 cells (1-2×10^4^/well) were loaded on slides, fixed with 4% formaldehyde. After pretreating with protease K (2 μg/mL), glycine and acetic anhydride, the slides were pre-hybridized for 60 min and hybridized using 300 ng/mL probes (250 μL) at 42°C against PCAT6. Lastly, slides were stained with PBS with DAPI (Solarbio, Beijing, China) and photographed with a fluorescence microscope.

### Western Blot Analysis

LNCaP and NCI-H660 cells were harvested using radioimmunoprecipitation assay (RIPA) Lysis (Elabscience, Wuhan, China), and protein concentration were detected by the BCA protein assay kit (Pierce, Rockford, IL, USA). 10 µg of protein were separated by 10% SDS-PAGE gels, followed by electrotransfer onto PVDF membranes (Arkema, Paris, France). The membrane was incubated with primary antibody against ChgA (1:1000, ab254557, Abcam), NSE (1:100, ab105389, Abcam), SYP (1:1000, ab32127, Abcam), Hnrnpa2b1 (5 µg/ml, ab31645, Abcam), and β-actin (1:1000, 3700, CST, Danvers, MA, USA) overnight at 4 °C, HRP-conjugated secondary antibodies (1:1000; ab205718; Abcam), and visualized through the ECL imaging system (Bio-Rad, Hercules, CA, USA).

### Cell Proliferation Assay

A Cell Counting Kit-8 (CCK-8, Dojindo, Japan) was applied to detect cell proliferation ability as instructed by the manufacturer. The transfected LNCaP or NCI-H660 cells were plated into 24-well plates (6×10^4^ cells/well) for 24, 48, and 72 h. 10 μL CCK-8 solution was added into per well followed by incubation for 2 hours. Lastly, the absorbance of the resulting solution was tested at 450 nm with a microplate reader (REAGEN, USA).

### Transwell

Transwell chamber (24-well) with Matrigel was applied to determine cell invasion following the manufacturer’s instructions. Briefly, 200 μl transfected LNCaP or NCI-H660 cells (3.0×10^5^) were added in the upper chamber with RPMI-1640 medium without serum. 500 μl RPMI-1640 medium contained 10% FBS was added to the lower chamber. After incubating for one day at 37 °C with 5% CO_2_, the cells on the lower surface of membranes were fixed with absolute ethanol, stained by 0.1% crystal violet for 15 min. The invasive cell number was counted using a Zeiss Microscope (Oberkohen, Baden-Wurttemberg, Germany).

### RNA Pull-Down Assay

After centrifuging at 12,000 g at 4 °C for 12 minutes, the LNCaP cell supernatant was harvested. Then, RIP buffer containing biotin-labeled PCAT6 was mixed with cell lysates for 60 minutes. Next, streptavidin beads were added to each binding reaction followed by incubation for 60 minutes. Lastly, the protein in the elution complex was determined with western blot assay.

### Luciferase Assay

The wildtype (WT) or mutant (Mut) PCAT6 promoters were constructed into PGL3-basic plasmid (YouBia, Hunan, China). LNCaP and NCI-H660 cells were seeded into a 24-well plate followed by transfection with PGL3-Luc supplemented with WT or Mut PCAT6 promoters with Lipofectamine (Invitrogen) and pRL-TK was applied as control. Luciferase activity was examined using the Dual-Luciferase^®^ Reporter Assay system (Promega Corporation, Madison, WI, USA) as the manufacturer’s protocol.

### Xenograft in Nude Mice

BALB/c nude mice (male; 4-6 w; 18-22 g) were selected from Shanghai Model Organisms Center, Inc (China). The animal experiment was approved by the Ethics Committee of the Tongji Hospital, Tongji University of Medicine. The mice were injected with the PCAT6 lentivirus vector. Briefly, LNCaP cells were infected with lentiviral vectors with a titer of 2*10^8^ plaque-forming units (PFUs)/mL. After obtaining stably transfected cell lines, cell suspensions (5*10^7^ cells/mL) were injected intravenously into the nude mice’s tail vein. The tumor growth was measured at specific times (7th, 14th, 28th, and 35th) after inoculation.

### Statistical Analysis

Statistical analysis was carried out with the SPSS 17.0 software (IBM-SPSS, Inc., Chicago, IL, USA). Each test process was independently repeated 3 times. Results are presented as mean ± SD. Comparison of two groups was identified by t-test analysis, or one-way analysis of variance (ANOVA) followed by the Scheffé test. A P-value of < 0.05 was considered statistically significant.

## Results

### PCAT6 Expression Was Increased in NEPC Cells and Tissues

**Figure 1:**
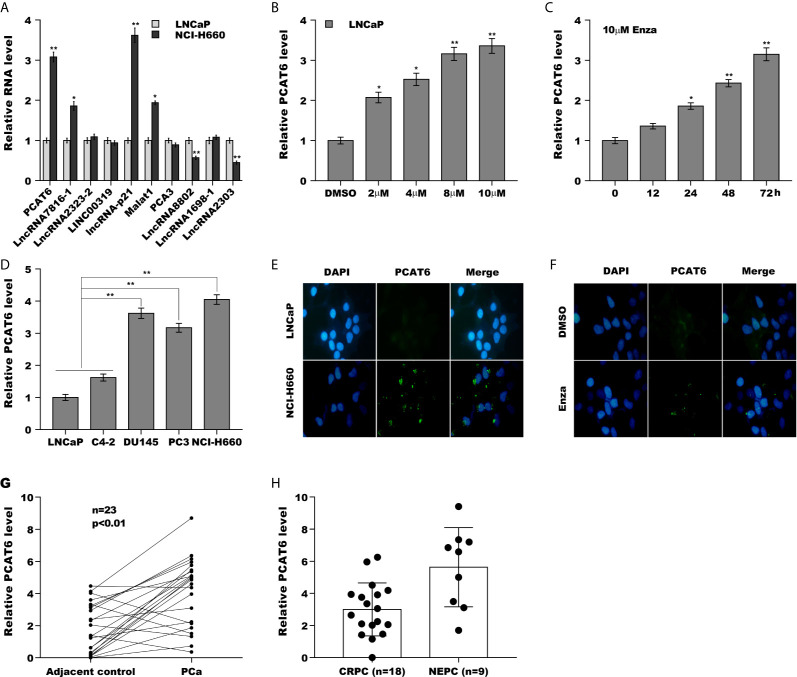
PCAT6 expression was increased in NEPC cells and tissues. **(A)** qPCR analysis of the top 10 lncRNAs (PCAT6, LncRNA7816-1, LncRNA2323-2, LINC00319, lncRNA-p21, Malat1, PCA3, LncRNA8802, LncRNA1698-1 and LncRNA2303) mRNA expression in NCI-H660 cells and LNCaP cells. **(B)** qPCR analysis of PCAT6 mRNA expression in NE-like cells (PC3, DU145, and NCI-H660) and LNCaP and C4-2 cells. **(C, D)** The mRNA expression of PCAT6 was determined in LNCaP cells treated with different Enza concentration (2 μM, 4 μM, 8 μM, 10 μM) for 72 h or 10 μM for different times (0 h, 12 h, 24 h, 48 h, 72 h). **(E)** Fluorescent *in situ* hybridization (FISH) assay of the PCAT6 expression in LNCaP and NCI-H660 cells. **(F)** Fluorescent *in situ* hybridization (FISH) assay of the PCAT6 expression in LNCaP cells with 10 μM Enza treatment for 72 h. **(G)** qPCR analysis of PCAT6 expression in Pca tissues and adjacent normal tissues. **(H)** qPCR analysis of PCAT6 expression in NEPC (n=9) and CRPC (n=18) samples. *p<0.05. **p<0.01.

Luo et al., has found that the expression of lncRNAs is dys-regulated in NEPC samples and Enzalutamide enhances lncRNA-p21 expression to facilitate NED of PCa (**Supporting Figure S1A** and **Supporting Table S1**) (20). Here the top 10 lncRNAs significantly dys-regulated in NEPC samples were reassessed in androgen-sensitive LNCaP cells and NE-like NCI-H660 cells. As shown in **Figure 1A**, PCAT6 was identified as the most highly expressed lncRNA except lncRNA-p21 in NCI-H660 cells compared with LNCaP cells. The PCAT6 level in NE-like cells (PC3, DU145, and NCI-H660) was commonly increased compared with LNCaP as well as C4-2 cells (**Figure 1B**), indicating the potential effect of PCAT6 on NED. We then investigated the effect of treatment time and concentration of Enzalutamide on PCAT6 expression in LNCaP cells. **Figures 1C, D** revealed that Enzalutamide treatment significantly enhanced PCAT6 level in a dose-dependent and time-dependent fashion. The results from RNA-FISH also indicated that PCAT6 expression was higher in NCI-H660 cells when compared with LNCaP cells (**Figure 1E**), and Enzalutamide treatment upregulated PCAT6 level in LNCaP cells (**Figure 1F**). We then assessed PCAT6 level in human PCa tissues and paired normal tissues. **Figure 1G** revealed that PCAT6 expression was markedly increased in most tumor tissues compared to controls. Furthermore, the PCAT6 level was markedly upregulated in NEPC tissues compared with CRPC tissues (**Figure 1H**), indicating that upregulated PCAT6 was correlated with PCa NED.

### PCAT6 Promoted NED, Proliferation, and Invasion of PCa Cells *In Vitro*

**Figure 2:**
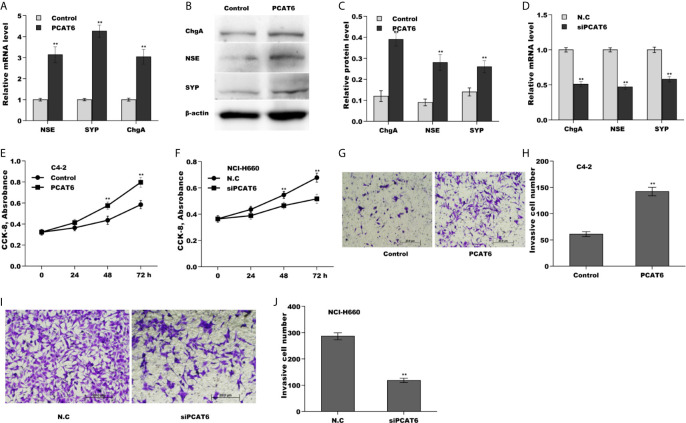
PCAT6 promoted NED, proliferation, and invasion of PCa cells *in vitro*. **(A)** qPCR analysis of NE markers (NSE, SYP, and ChgA) mRNA expression in LNCaP cells after PCAT6 overexpression. **(B, C)** Western blot analysis for NSE, SYP, and ChgA protein level in LNCaP cells after PCAT6 overexpression. **(D)** qPCR analysis of NSE, SYP, and ChgA mRNA expression in NCI-H660 cells after PCAT6 knockdown. **(E)** LNCaP cell proliferation was assayed using CCK-8 after PCAT6 overexpression. **(F)** NCI-H660 cell proliferation was assayed using CCK-8 after PCAT6 knockdown. **(G, H)** LNCaP cell invasion was assessed using transwell invasion assay after PCAT6 overexpression. **(I, J)** NCI-H660 cell invasion was assessed using transwell invasion assay after PCAT6 knockdown. *p<0.05. **p<0.01.

To explore the biological function of PCAT6 on PCa cells, PCAT6 was overexpressed in LNCaP cells or inhibited in NCI-H660 cells and then NED, proliferation, invasion of PCa cells were assessed. Forced expression of PCAT6 remarkably enhanced mRNA and protein levels of NE markers (NSE, SYP, and ChgA) (**Figures 2A–C**). On the contrary, PCAT6 knockdown significantly repressed the expression of NE markers (**Figure 2D**), indicating that PCAT6 positively regulated NED of PCa cells. Additionally, the effect of PCAT6 in regulating PCa cell proliferation and invasion was assessed. **Figures 2E, F** showed that PCAT6 overexpression promoted LNCaP cell proliferation, whereas PCAT6 knockdown repressed NCI-H660 cell proliferation. As expected, PCAT6 overexpression facilitated LNCaP cell invasion, whereas PCAT6 knockdown significantly suppressed NCI-H660 cell invasion (**Figures 2G–J**).

### PCAT6 Promoted PCa Growth and Metastasis *In Vivo*

**Figure 3:**
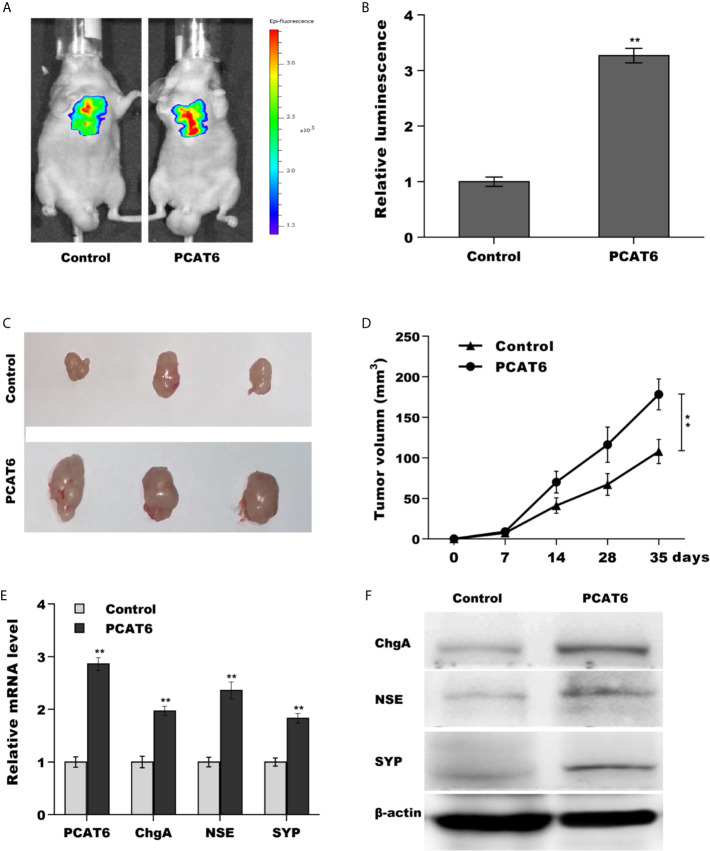
PCAT6 promoted PCa growth and metastasis *in vivo*. **(A, B)** Cancer metastasis was assayed using bioluminescent imaging after PCAT6-overexpressed. **(C, D)** LNCaP cells stably expressed with PCAT6 were injected intravenously into the tail vein of male nude mice and tumor volume was calculated at the different time points (7, 14, 28, 35 days). **(E)** qPCR analysis of PCAT6, NSE, SYP, and ChgA mRNA expression in LNCaP cells after PCAT6 overexpression. **(F)** Western blot analysis for NSE, SYP, and ChgA protein level in LNCaP cells after PCAT6 overexpression. **p<0.01.

Then, we investigated the role of PCAT6 in tumor growth and metastasis *in vivo*. To construct the murine metastatic PCa model *in vivo*, PCAT6-overexpressed LNCaP cells stably expressed luciferase were injected intravenously into tail vein of male nude mice and the bioluminescent signal was assessed at 10 weeks following injection. PCAT6 overexpression enhanced the bioluminescent signals, indicating the role of PCAT6 in promoting PCa metastasis (**Figures 3A, B**). To assess the effect of PCAT6 on tumor growth, PCAT6-overexpressed LNCaP cells were injected subcutaneously into nude mice and tumor growth was surveyed. **Figures 3C, D** showed that PCAT6 overexpression significantly promoted tumor growth at different time points. More importantly, the mRNA and protein expressions of NE markers in PCAT6-overexpressed groups were markedly upregulated compared to the control group (**Figures 3E, F**).

### PCAT6 Functioned as a ceRNA and Sponged miR-326

**Figure 4:**
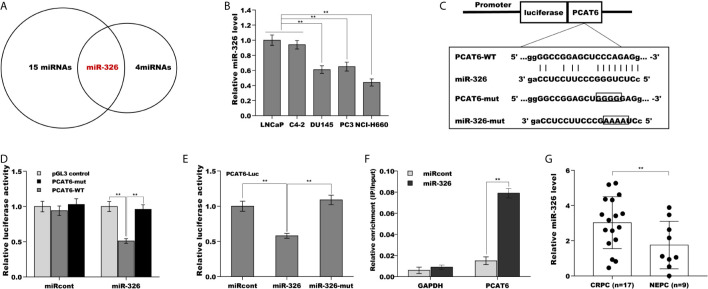
PCAT6 functioned as a ceRNA and sponged miR-326. **(A)** Starbase v2.0 database was used to predict potential miRNA binding sites in PCAT6. **(B)** qPCR analysis of miR-326 mRNA expression in NE-like cells (PC3, DU145, and NCI-H660) and LNCaP and C4-2 cells. **(C)** Schematic representation of the miR-326 site in PCAT6-3’UTR. **(D)** Luciferase activity was assayed in LNCaP cells co-transfected with miR-326 and luciferase reporters containing PCAT6-WT or PCAT6-Mut. Data are presented as the relative ratio of firefly luciferase activity to renilla luciferase activity. **(E)** Luciferase activity was assayed in LNCaP cells co-transfected with luciferase reporters containing PCAT6-WT and miR-326 or miR-326-Mut. **(F)** The direct binding of PCAT6 to miR-326 was affirmed using luciferase reporters in LNCaP cells treated with miR-326 or miR-326-mut. **(G)** qPCR analysis of miR-326 level in CRPC tissues (n=17) and NEPC tissues (n=9). **p<0.01.

Mounting evidence demonstrates that lncRNAs commonly function as ceRNA to absorb miRNAs, thus upregulate mRNA expression when lncRNAs are expressed in the cytoplasm (21, 22). The results from RNA-FISH indicated that PCAT6 was mainly located in the cytoplasm of PCa cells (**Figures 1E, F**), indicating that PCAT6 might act as a ceRNA. To identify the potential miRNAs sponged by PCAT6, the Starbase v2.0 database (http://starbase.sysu.edu.cn/) was applied to forecast potential miRNA binding sites in PCAT6 (23). The results from the bioinformatic analysis revealed that 15 miRNAs could be sponged by PCAT6 (**Supporting Table S2**). Among 15 miRNAs, miR-326 was selected for further analysis because miR-326 was verified as a biomarker for prostate cancer diagnosis (**Figure 4A**) (24, 25). Interestingly, the miR-326 level in NE-like cells was reduced compared with LNCaP and C4-2 cells (**Figure 4B**). To assess the direct combination of PCAT6 and miR-326, the recombinant plasmids of pGL3-PCAT6-WT and pGL3-PCAT6-Mut were constructed, co-transfected with miR-326 into LNCaP cells, respectively (**Figure 4C**). The results from the luciferase test revealed that pGL3-PCAT6-WT luciferase activity was markedly repressed by co-transfection with miR-326 compared with miRcont, whereas miR-326 did not affect the luciferase activity of pGL3-PCAT6-Mut (**Figure 4D**). Furthermore, miR-326 mutant lost its suppressor role in regulating luciferase activity of pGL3-PCAT6-WT (**Figure 4E**). RNA pull-down was performed to further validate the direct combination of PCAT6 and miR-326. As shown in **Figure 4F**, PCAT6 was more enriched in the miR-326 compared with miRcont. miR-326 was lowly expressed in small cell carcinoma tissues compared to CRPC tissues (**Figure 4G**). These data suggest that PCAT6 functioned as a tumor activator in PCa progression, at least in part by sponging miR-326.

### PCAT6 Activated hnRNPA2B1 Expression in miR-326-Dependent Manner

**Figure 5:**
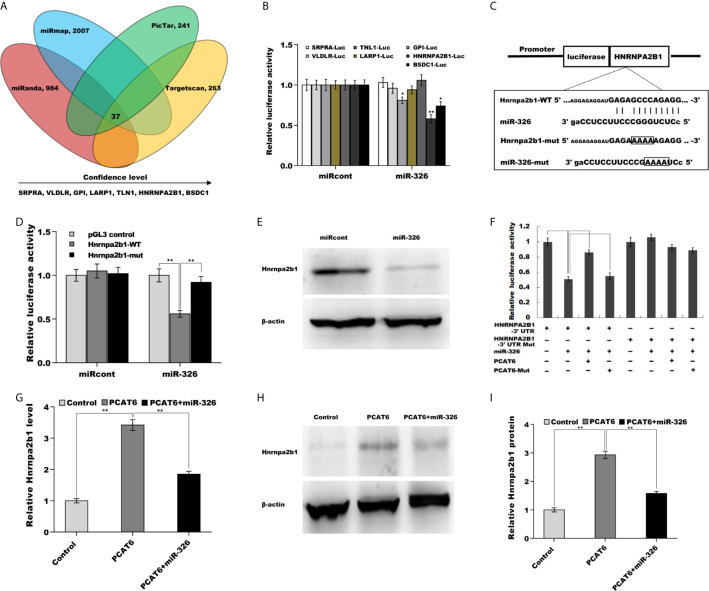
PCAT6 activated Hnrnpa2b1 expression in miR-326-dependent manner. **(A)** Four bioinformatics algorithms (miRanda, miRmap, picTar, and TargetScan) were used to predict the common target genes of miR-326. **(B)** Luciferase activity was assayed in NCI-H660 cells co-transfected with luciferase reporters containing SRPRA-WT, VLDLR-WT, TNL1-WT, LARP1-WT, GPI-WT, HNRNPA2B1-WT, or BSDC1-WT and miR-326 or miR-326-Mut. **(C)** Schematic representation of the miR-326 site in Hnrnpa2b1-3’UTR. **(D)** Luciferase activity was assayed in NCI-H660 cells co-transfected with miR-326 and luciferase reporters containing Hnrnpa2b1-WT or Hnrnpa2b1-Mut. **(E)** Western blot analysis for Hnrnpa2b1 protein level in NCI-H660 cells after miR-326 overexpression. **(F)** Luciferase activity was assayed in NCI-H660 cells co-transfected with luciferase reporters containing Hnrnpa2b1-3’ UTR, Hnrnpa2b1-3’ UTR-Mut, PCAT6-WT, or PCAT6-Mut. **(G)** qPCR analysis of Hnrnpa2b1 mRNA expression in NCI-H660 cells after PCAT6 overexpression in the presence or absence of miR-326. **(H, I)** Western blot analysis for Hnrnpa2b1 protein level in NCI-H660 cells after PCAT6 overexpression in the presence or absence of miR-326. **p<0.01.

We next identified the target gene of miR-326 in PCa cells. To this end, four bioinformatics algorithms (miRanda, miRmap, picTar, and TargetScan) were used to predict the commonly potential targets. The results from bioinformatics analysis revealed that 37 genes were the common target genes of miR-326 (**Figure 5A** and **Supporting Table S3**). Among them, *hnRNPA2B1* was identified as the most significant target through luciferase activity assay (**Figure 5B**). To verify the statement, the recombinant plasmids of pGL3-hnRNPA2B1-3’UTR-WT and its mutant (pGL3-hnRNPA2B1-3’UTR-Mut) was constructed through inserting hnRNPA2B1-3’UTR cDNA or its mutant into pGL3 vector (**Figure 5C**). The results from luciferase test revealed that pGL3-hnRNPA2B1-3’UTR-WT luciferase activity was markedly suppressed by co-transfection with miR-326 compared with miRcont, whereas miR-326 did not affect pGL3-hnRNPA2B1-3’UTR-Mut luciferase activity (**Figure 5D**). These data suggested that miR-326 could straightway bind hnRNPA2B1-3’UTR by their own miRNA binding sites. Furthermore, miR-326 overexpression markedly repressed the protein level of hnRNPA2B1 in NCI-H660 cells (**Figure 5E**). The regulatory correlations of PCAT6, miR-326, and Hnrnpa2b1 were next assessed. **Figure 5F** revealed that pGL3-hnRNPA2B1-3’UTR luciferase activity was suppressed due to miR-326 overexpression, but the effect was remitted by transfecting pGL3-PCAT6-WT. pGL3-PCAT6-Mut did not affect the potential of miR-326 to repress the luciferase activity of pGL3-hnRNPA2B1-3’UTR, showing that PCAT6 competed with hnRNPA2B1 to binding to miR-326 and that PCAT6 functioned as an endogenous “sponge” by absorbing miR-326. Given that PCAT6 sponged miR-326 and miR-326 directly repressed hnRNPA2B1, we speculated that PCAT6 might enhance hnRNPA2B1 expression in miR-326-dependent manner. As expected, PCAT6 enhanced the mRNA and protein level of hnRNPA2B1 in NCI-H660 cells, whereas miR-326 overexpression markedly repressed the effect (**Figures 5G–I**).

### The Role of PCAT6/miR-326/hnRNPA2B1 Axis in Regulating PCa Cells Proliferation, Invasion, and NED

**Figure 6:**
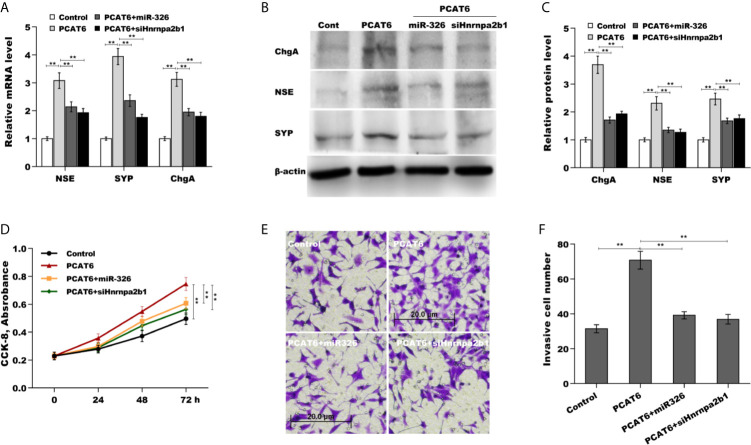
The role of PCAT6/miR-326/Hnrnpa2b1 axis in regulating PCa cells proliferation, invasion, and NED. **(A)** qPCR analysis of NSE, SYP, and ChgA mRNA expression in LNCaP cells after PCAT6 overexpression in the presence or absence of miR-326 overexpression or Hnrnpa2b1 knockdown. **(B, C)** Western blot analysis for NSE, SYP, and ChgA protein level in LNCaP cells after PCAT6 overexpression in the presence or absence of miR-326 overexpression or Hnrnpa2b1 knockdown. **(D)** LNCaP cell proliferation was assayed using CCK-8 after PCAT6 overexpression in the presence or absence of miR-326 overexpression or Hnrnpa2b1 knockdown. **(E, F)** LNCaP cell invasion was assessed using transwell invasion assay after PCAT6 overexpression in the presence or absence of miR-326 overexpression or Hnrnpa2b1 knockdown. **p<0.01.

Finally, we examined the role of PCAT6/miR-326/hnRNPA2B1 axis in regulating PCa cells proliferation, invasion, and NED. As shown in **Figures 6A–C**, PCAT6 enhanced the mRNA and protein level of NE markers in LNCaP cells compared to control group, whereas both miR-326 overexpression and hnRNPA2B1 knockdown significantly reversed the effect. PCAT6 promoted LNCaP cells proliferation and invasion, whereas both miR-326 overexpression and hnRNPA2B1 knockdown significantly repressed the effect of PCAT6 on pro-proliferation and pro-invasion (**Figures 6D–F**). All results indicate that PCAT6 promoted Enzalutamide-induced PCa NED by regulating miR-326/hnRNPA2B1 axis.

## Discussion

ADT is the standard indication for treating locally advanced and metastatic PCa, achieved primarily by GnRH (gonadotropin-releasing hormone) agonists (26, 27), GnRH antagonists (28, 29), and androgen receptor antagonistic agents (30). Currently, the most widely used anti-androgen drugs are bicalutamide, Abiraterone and Enzalutamide (4). To some extent, Enzalutamide treatment prolongs the survival time of PCa patients, but Enzalutamide would also lead to a series of side effects including drug resistance, cancer metastasis and neuroendocrine differentiation of prostate cancer (5, 6). However, the underlying mechanism of neuroendocrine differentiation remains unclear. In this study we demonstrate that (i) PCAT6 level is increased in NEPC cells and tissues, (ii) Upregulated PCAT6 facilitates NED, proliferation, invasion of PCa cells *in vitro* and *in vivo*, (iii) PCAT6 acts as a ceRNA and sponges miR-326 to de-repress Hnrnpa2b1 expression, (iv) PCAT6 promotes PCa cells proliferation, invasion, and NED by regulating miR-326/Hnrnpa2b1 axis. All results identify the regulatory role of PCAT6/miR-326/Hnrnpa2b1 axis in PCa process and might provide a potential target for PCa therapy.

PCAT6, lied on chromosome 1q32.1, functions as a tumor activator in multiple types of cancers like non-small-cell lung cancer (12), osteosarcoma  (31), gastric cancer (32), and hepatocellular carcinoma (33). For instance, Bai et al., reported that PCAT6 facilitates gastrointestinal stromal cancer cell stemness and proliferation by adsorbing miR-143-3p and thus Wnt signaling pathway activation (34). In triple-negative breast cancer, PCAT6 inhibition increases radiosensitivity of cancer cells through ceRNA pattern to regulating miR-185/TPD52 axis (35). PCAT6 also enhances chemoresistance to cisplatin in cervical cancer by sponging miR-543 and activating ZEB1 expression (36). Although a previous study has identified the significant increase of PCAT6 expression in PCa tissues (20), the biological function of PCAT6 on PCa progression is unclear. In the study, we demonstrated that PCAT6 is highly expressed in NEPC tissues and cells. Enza treatment induces a significant upregulation of PCAT6 expression PCa cells. Forced expression of PCAT6 promotes proliferation, invasion, and NED of PCa *in vitro* and *in vivo*, while PCAT6 inhibition suppresses PCa cell proliferation, invasion, and NED.

Previous studies have revealed that PCAT6 generally functions as a ceRNA to exert its biological roles. Here we first assessed the subcellular localization of PCAT6 in PCa cells, and data showed that PCAT6 is mainly located in cytoplasm of PCa cells, indicating that PCAT6 might function as a ceRNA in PCa. Actually, the results from bioinformatics analysis and experimental studies demonstrate that PCAT6 functions as ceRNA by absorbing miR-326, thus leading to a de-suppression of Hnrnpa2b1 target gene. Functionally, both miR-326 overexpression and Hnrnpa2b1 knockdown effectively reverses PCAT6-induced PCa cells proliferation, invasion, and NED.

Although Enzalutamide significantly extends the survival of PCa patients, Enzalutamide treatment also results in a series of side effects such as drug resistance and NED of PCa (5, 6). It is essential to reveal the mechanism underlying Enzalutamide resistance. In a previous study we demonstrated that lncRNA-p21 level is increased after Enzalutamide treatment and promotes NED of PCa through regulating Enhancer of Zeste Homolog 2/signal transducer and activator of transcription 3 signaling (20). Besides lncRNA-p21, a large amount of lncRNAs (PCAT6, PCAT1, Malat1, etc.) are abnormally expressed. Similarly to lncRNA-p21, here we further demonstrated that PCAT6 facilitates PCa NED by regulating miR-326/HNRNPA2B1. The role miR-326 in PCa progression is definite. Liang et al. revealed that miR-326 exerts a tumor suppressor gene in human PCa through repression Mucin1 (37). Huang et al. reported that miR-326 represses PCa cell growth and invasion through targeting Mitogen-Activated Protein Kinase 1 (MAPK1) (38). The limitations of the study are whether lncRNA-p21 and PCAT6 synergistically facilitate PCa NED. In summary, the current data demonstrate that Enza-induced increase of PCAT6 expression promotes prostate cancer neuroendocrine differentiation by regulating miR-326/Hnrnpa2b1 axis.

## Data Availability Statement

The datasets presented in this study can be found in online repositories. The names of the repository/repositories and accession number(s) can be found in the article/**Supplementary Material**.

## Ethics Statement

The animal study was reviewed and approved by The Tongji Hospital, Tongji University of Medicine.

## Author Contributions

All authors contributed to the article and approved the submitted version.

## Funding

The project was funded by the National Natural Science Foundation of China (No. 81802521).

## Conflict of Interest

The authors declare that the research was conducted in the absence of any commercial or financial relationships that could be construed as a potential conflict of interest.
