# Supplementary figures and images for: Enzalutamide-Induced Upregulation of PCAT6 Promotes Prostate Cancer Neuroendocrine Differentiation by Regulating miR-326/HNRNPA2B1 Axis
Source: Front Oncol. 2021 Jun 30;11:650054. doi: 10.3389/fonc.2021.650054 (PMC8278330; doi:10.3389/fonc.2021.650054)

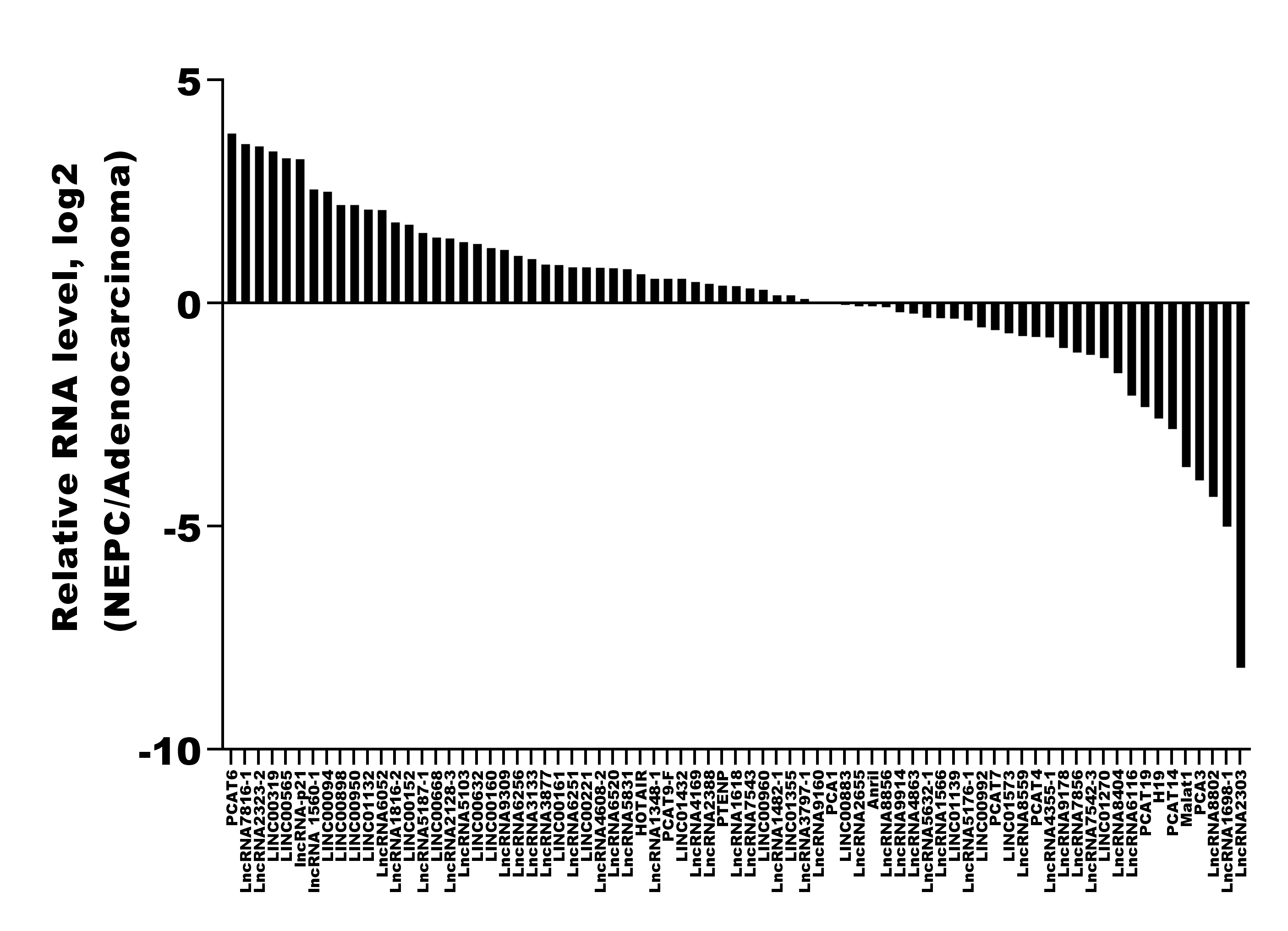

Supplement: Supplementary file 1 [file Image_1.tif]

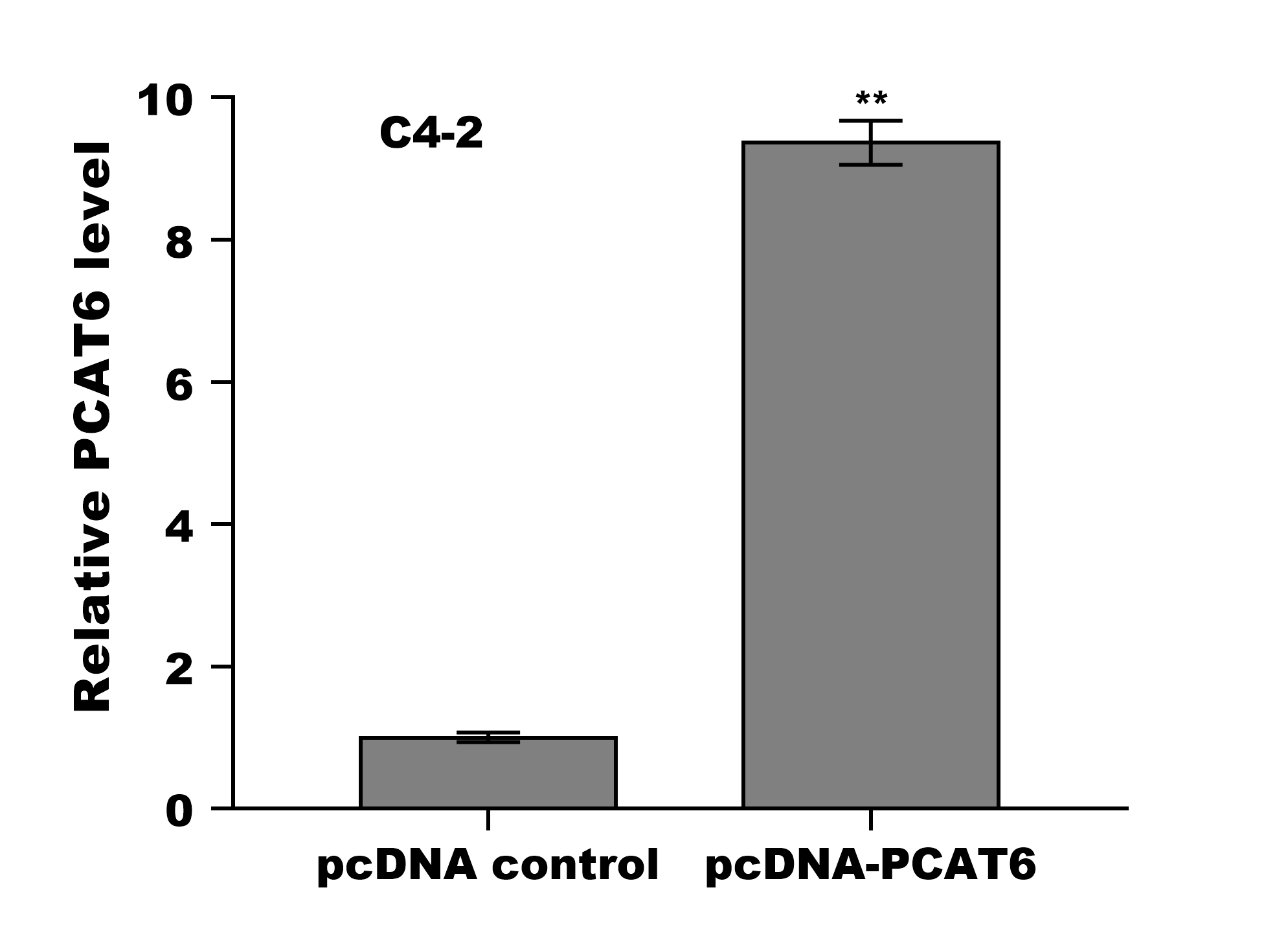

Supplement: Supplementary file 2 [file Image_2.tif]

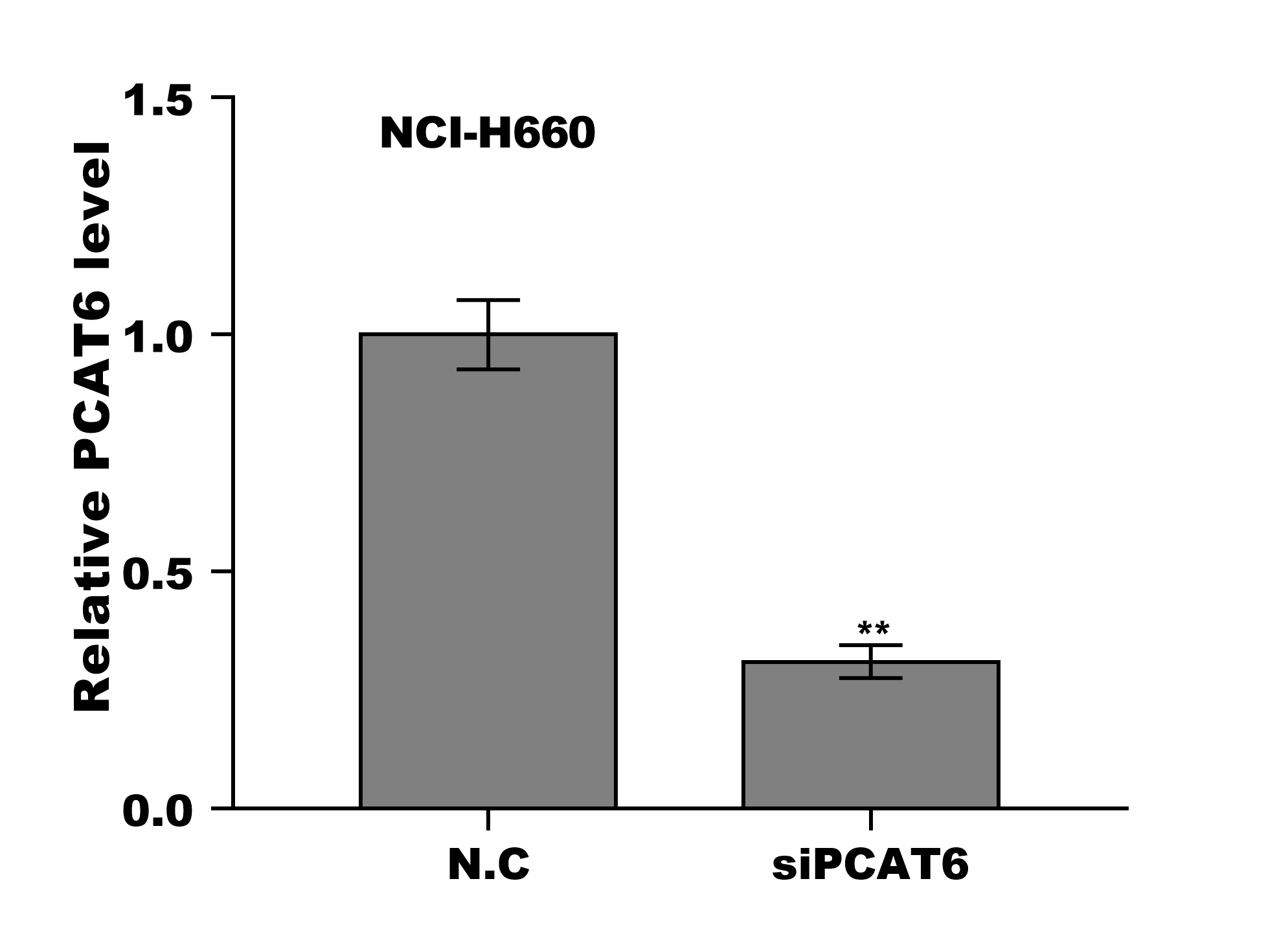

Supplement: Supplementary file 3 [file Image_3.tif]
