# Supplementary material for: Enzalutamide-Induced Upregulation of PCAT6 Promotes Prostate Cancer Neuroendocrine Differentiation by Regulating miR-326/HNRNPA2B1 Axis
Source: Front Oncol. 2021 Jun 30;11:650054. doi: 10.3389/fonc.2021.650054 (PMC8278330; doi:10.3389/fonc.2021.650054)
Supplement: Supplementary file 4 [file Table_1.docx]

| lncRNAs | NEPC vs. Adenocarcinoma (Fold, log2) |
| --- | --- |
| PCAT6 | 13.9288 |
| lncRNA7816-1 | 11.8762 |
| lncRNA2323-2 | 11.4716 |
| LINC00319 | 10.5561 |
| LINC00565 | 9.5137 |
| LncRNA-p21 | 9.3827 |
| lncRNA1560-1 | 5.8563 |
| LINC00094 | 5.6569 |
| LINC00898 | 4.5948 |
| LINC00950 | 4.5948 |
